# Understanding Antiretroviral Therapy Adherence in Children With HIV: A Review of Barriers and Strategies

**DOI:** 10.1155/arat/1760508

**Published:** 2026-02-08

**Authors:** Flora Ramona Sigit Prakoeswa, Faradiba Maharani, Purista Tiara Dewi, Azka Hafiy Sulistiyo, Rochmadina Suci Bestari, Dodik Nursanto, Listiana Masyita Dewi

**Affiliations:** ^1^ Dermatovenereology Department, Faculty of Medicine, Universitas Muhammadiyah Surakarta, Central Java, Indonesia, ums.ac.id; ^2^ Dermatovenereology Department, PKU Muhammadiyah Hospital, Surakarta, Indonesia; ^3^ Faculty of Medicine, Universitas Sebelas Maret, Central Java, Indonesia, uns.ac.id; ^4^ Anatomy and Embryology Department, Faculty of Medicine, Universitas Muhammadiyah Surakarta, Central Java, Indonesia, ums.ac.id; ^5^ Faculty of Medicine, Universitas Muhammadiyah Surakarta, Central Java, Indonesia, ums.ac.id; ^6^ Molecular Biology Department, Faculty of Medicine, Universitas Muhammadiyah Surakarta, Central Java, Indonesia, ums.ac.id

**Keywords:** adherence, antiretroviral therapy (ART), barriers, children, pediatric HIV

## Abstract

**Introduction:**

Despite advancements in antiretroviral therapy (ART) availability, maintaining optimal adherence remains difficult, especially in low‐ and middle‐income countries (LMICs). This literature review synthesized evidence on barriers and interventions influencing adherence to ART among children and adolescents with HIV, focusing on studies conducted in LMICs, particularly in Africa and Asia, where adherence and challenges are more prevalent.

**Methods:**

This narrative review draws on peer‐reviewed studies to identify barriers to ART adherence, such as medication complexity, caregiver‐related issues, socioeconomic factors, and healthcare infrastructure limitations. A thorough search was conducted using electronic databases like PubMed, Google Scholar, and Scopus, covering relevant primary and secondary literature from 2010 to 2023 in LMICs in Africa and Asia. It also evaluates the effectiveness of digital tools and psychosocial interventions in enhancing adherence to ART among children with HIV.

**Results and Discussion:**

Findings reveal that adherence remains suboptimal, with rates ranging from 62% to 95% across settings. Major barriers include regimen complexity, psychosocial stressors, and limited healthcare access, while interventions such as digital adherence tools, caregiver education, and community‐based programs demonstrate measurable improvements. Evidence highlights that targeted interventions can improve outcomes. Family support programs in Uganda increased adherence from 66% to 83%, and SMS reminder systems improved adherence by up to 5.5% among adolescents. Nevertheless, recent reductions in international funding pose a substantial threat to the sustainability of these gains in resource‐limited settings.

**Conclusion:**

Improving adherence among children and adolescents requires not only simplified regimens and psychosocial support but also sustainable financing mechanisms. Future strategies must anticipate barriers related to funding cuts, drug resistance, and the transition from pediatric to adult care.

## 1. Introduction

HIV/AIDS continues to be a serious global health issue, especially among children, who are particularly vulnerable [1, 2]. As of 2023, an estimated 2.38 million children aged 0–18 were living with HIV worldwide, highlighting the ongoing need for effective treatment and care [3]. Antiretroviral therapy (ART) has been crucial in improving health outcomes for people living with HIV, including children populations, by reducing morbidity and mortality rates [4]. However, achieving optimal adherence to the ART regimen remains a significant challenge, which is necessary to control the virus and prevent drug resistance, which could lead to treatment failure [5, 6].

Despite improvements in ART availability, only about 57% of children aged 0–14 living with HIV received treatment in 2023, leaving many children at risk of disease progression [1]. Additionally, about 685 new HIV infections are reported in children each day, and approximately 250 children die daily from AIDS‐related causes [3]. These statistics show the urgent need for strategies that not only increase ART coverage but also focus on improving adherence, especially in low‐ and middle‐income countries (LMICs) where the burden is highest.

One key difference between children and adults living with HIV is that children rely heavily on their caregivers for treatment adherence [4]. Unlike adults, children cannot manage their medication on their own, meaning that parents, guardians, or healthcare providers play a crucial role in ensuring they take their medication regularly. Caregivers are responsible for managing daily doses, handling side effects, and navigating healthcare systems [7]. The mental health, social support, and knowledge of these caregivers can directly impact how well children adhere to ART [7]. In many low‐resource settings, caregivers face additional challenges such as stigma, lack of healthcare access, and financial struggles, which can further hinder adherence [8, 9].

As children grow into adolescents, they begin to take on more responsibility for their own healthcare. However, this transition often brings new challenges. Adolescents may face medication fatigue, peer pressure, and the emotional difficulties of living with HIV, which can lead to a decline in adherence [4, 8, 10]. Therefore, interventions need to not only focus on children but also provide caregivers with support to encourage adherence through different stages of childhood and adolescence.

New digital tools, like mobile health apps, electronic medication reminders, and telemedicine, have shown potential in helping children and their caregivers stick to ART, particularly in high‐resource settings [11, 12]. These technologies offer real‐time monitoring and reminders, which can help both children and caregivers manage treatment more effectively [11]. However, in regions where healthcare infrastructure is limited, stigma is prevalent, and there are economic barriers, implementing these tools can be challenging [13]. Understanding these barriers and exploring ways to support adherence in different regions is crucial for creating effective, targeted interventions. In addition to technological solutions, maintaining high adherence to ART in children is complicated by several psychosocial factors, such as mental health challenges and family dynamics [14, 15].

Over the past decade, the landscape of pediatric ART has changed significantly with the introduction of simplified formulations such as fixed‐dose combinations (FDCs) [16]. These advances have improved tolerability and reduced pill burden, making adherence easier for children and adolescents compared to earlier regimens. As a result, this review places particular emphasis on literature published after 2014, while earlier studies are included primarily for historical context.

This review focuses on evidence from LMICs, particularly in Africa and Asia, where adherence challenges remain most pronounced. Findings from high‐income countries such as the United States and Europe are not emphasized, as their healthcare systems, broader drug availability, and funding structures create contexts that are less generalizable to LMIC settings.

This literature review aims to explore existing research on ART adherence in children with HIV, focusing on the factors that influence adherence and the role of caregivers. It also highlights interventions tested in LMIC contexts and examines how findings differ across age groups, with special attention to the unique challenges faced during adolescence. By identifying effective methods for both age groups, this review hopes to contribute to the development of comprehensive support systems that can improve adherence and health outcomes for children living with HIV.

## 2. Methods

This narrative review synthesizes current research on ART adherence in pediatric populations, focusing on factors that influence adherence, the role of caregivers, and interventions designed to support adherence. We conducted a search of peer‐reviewed studies using electronic databases, including PubMed, Google Scholar, and Scopus, covering the years 2010–2023, with particular emphasis on studies published after 2014, when simplified formulations such as FDCs became widely available.

The following keywords and combinations of Medical Subject Headings (MeSH) terms were used: “pediatric HIV,” “ART adherence,” “digital adherence tools,” “caregiver support,” “psychosocial interventions,” “adolescent HIV treatment,” and “low‐resource settings.”

### 2.1. Inclusion and Exclusion Criteria

In this review, we included studies that focused on children and adolescents aged 0–18 living with HIV who were receiving ART. Priority was given to studies conducted in LMICs, particularly in Africa and Asia, where adherence challenges are most prevalent. We prioritized research that explored factors influencing ART adherence, including caregiver involvement, psychosocial elements, and technological innovations such as mobile health solutions. Additionally, studies that reported on interventions aimed at improving adherence, particularly in low‐resource settings, were included to provide insights into practical strategies for enhancing ART adherence in pediatric populations. Only studies published in peer‐reviewed journals or reputable organizational reports were considered for this review.

We excluded studies that focused solely on adult populations or did not provide specific data related to ART adherence in pediatric groups. Opinion pieces, reviews, editorials, and studies lacking empirical evidence were also excluded to ensure the review’s focus on high‐quality, actionable research. The study used in this review is summarized in Table 1 for factors influencing adherence, Table 2 for technological innovations, and Table 3 for community intervention, caregiver support, and peer group support.

**Table 1:** Selected study used to discuss about factors influencing ART adherence.

No	Study	Design	Population	Adherence rates	Factors influencing adherence	Interventions	Region	Key findings
1	Prasitsuebsai et al. (2018)	Prospective, observational cohort study	Adolescents (12–18), 309 participants	< 80% adherence	Pill fatigue, long‐term treatment burden, and stigma	Audio computer‐assisted self‐interview (ACASI)	Thailand, Vietnam, Malaysia	Sexual and substance use risk behaviors and ART adherence
2	Bhattacharya et al. (2011)	Cross‐sectional study	Children (< 15), 90 participants	91.4% adherence	Increasing duration of ART, uneducated caregiver, orphan, efavirenz‐based regimen, female	Caregiver interviews using structured questionnaires.	India	Caregiver education > 5th grade improved adherence
3	Brittain et al. (2018)	Cohort design	Adolescents (9–14), 474 participants	70% adherence	Caregiver, gender, and patient age	Self‐report from both adolescents and their caregivers	South Africa	Adolescents with missed doses were significantly more likely to have elevated viral loads (VL ≥ 50 copies/mL).
4	Bermudez et al. (2016)	Randomized controlled trial (RCT)	Adolescents (10–16), 702 participants	70.6% adherence	Economic equity, demographic factors (age, gender, number of HIV medications), social equity	Baseline data collection, economic, and social equity measures	Uganda	Economic and social equity improved adherence
5	Nabunya et al. (2020)	Randomized clinical trial (RCT)	Adolescents (10–16), 702 participants	66% adherence	Sociodemographic and household characteristics, family factors, and adherence self‐efficacy	HIV Treatment Adherence Self‐Efficacy Scale, family cohesion scale, perceived child–caregiver support scale, child–caregiver communication scale	Southern Uganda	Underscore family as a microsystem for HIV‐infected adolescents in southern Uganda that provides both tangible and emotional support to enhance adherence self‐efficacy
6	Eticha et al. (2014)	Cross‐sectional study	Children (3 months–14 years old), 193 participants	83.4%–89.1% adherence	Age of child and caregivers, gender of child and caregivers, ethnic group, employment status, marital status, educational status, religion of caregiver, relation with child	A structured questionnaire to assess adherence to antiretroviral medications and its associated factors was adopted from other similar setups	Ethiopia	Caregivers are significant to improved adherence
7	Gemechu et al. (2023)	Cross‐sectional study	Children (5–14), 282 participants	87.2% adherence	Biological caregivers, residing in urban areas and being knowledgeable	A structured questionnaire to assess adherence to antiretroviral medications and its associated factors was adopted from other similar setups	Ethiopia	Adherence counseling targeting nonbiological parents and those who come from rural areas was recommended.
8	Nichols et al. (2019)	Site‐randomized clinical trial	Children and adolescent (7–18 years), 440 participants	93,2% adherence	Caregiver’s education level, children receiving help with medication from biological parents, proximity to the hospital	Three‐day recall using self‐reported questionnaire to caregiver, the child, and the pharmacy‐based time‐to‐refill of ART	Ghana	Nonadherence to antiretroviral therapy among undisclosed HIV‐infected children
9	Vreeman et al. (2015)	Prospective cohort study	Children (0–14 years), 191 participants	93% adherence	Child‐level factors, caregiver‐level barriers, social factors	Used MEMS to monitor adherence and evaluated caregiver‐reported questionnaire items to assess ART adherence.	US	Evaluation of caregiver‐reported antiretroviral therapy adherence
10	Lahai et al. (2020)	Cross‐sectional study	Children (< 14 years), 188 participants	5.9% adherence	Child‐related factors, caregiver‐related factors, and institutional factors	Highly active antiretroviral therapy (HAART)	Sierra Leone	High percentage of nonadherence among pediatric HIV‐infected/AIDS patients is caused by nondisclosure of HIV status and the involvement of nuclear family members.
11	Yi Siyan et al. (2018)	Cohort study	Adolescent (15–17 years)	95,4% adherence	Treatment fatigue, side effects, anticipated stigma, fear of HIV status disclosure, psychological factors, client–provider relationship, and inconsistent daily routine	Visual analog scale to assess adherence to ART	Cambodia	Adolescents living with HIV receiving care and treatment services in antiretroviral therapy
12	Ricci et al. (2016)	Cross‐sectional study	Children (1–12), 77 participants	≥ 95% adherence	Sociodemographic characteristics of the caregiver (age, female gender, race, income, household, use of alcohol, HIV +) and characteristics of the patient (gender, ARV formulation)	Caregivers’ education and reinforcement of the need for correct use of antiretrovirals	Brazil	There was a significant association between long‐term and 4‐day children adherence, as referred by caregiver
13	Yiryuo et al. (2024)	Phenomenological design	Adolescents (12–16 years), 455 participants, and children (2–14 years), 13 participants	62% adherence	Financial challenges, human‐related challenges. Challenges at healthcare centers, transportation issues, difficulties in disclosing children’s HIV status, medication‐related challenges	Seeking antiretroviral therapy services.	Ghana	Challenges and support experienced by family caregivers seeking antiretroviral therapy services
14	Nabukeera‐Barungi et al. (2015)	Convergent design involving both qualitative and quantitative data collection methods.	Adolescent, 1.824 participants	> 90% adherence	Stigma, discrimination, family situations, socioeconomic factors, medication issues, and challenges related to healthcare systems.	Antiretroviral therapy (ART)	Uganda	Adherence to antiretroviral therapy and retention in care
15	Arage et al. (2014)	Cross‐sectional study	Children (2 months ‐ 14 years old), 464 participants	≥ 95% adherence	Forgetfulness, child’s refusal to take the drugs, transportation problem, run out of pills, illness of the caregivers, pill burden, side effect of drug, illness of the child, taste of the drugs	Health care providers should educate caregivers about ART and the avoidance of substance use.	Ethiopia	Caregiver’s knowledge about antiretroviral therapy, no current use of substances, close proximity to health facilities, and letting child’s know his/her HIV status improves adherence to antiretroviral therapy.

**Table 2:** Selected studies to discuss technological innovations in adherence supports.

No	Study	Design	Population	Technological intervention	Findings	Outcomes
1	Willis et al. (2019)	RCT	Children (10–15), 47 participants	Community adolescent treatment supporters (CATS) intervention	Adolescents receiving CATS services reported a significant increase in linkage to health services and retention in care. While the intervention group showed increased linkage scores, the control group experienced a decline in retention. The intervention group had a significant improvement in adherence, increasing from 44.2% to 71.8%. Adolescents in the intervention group reported improved confidence, self‐esteem, and quality of life. Unexpectedly, the intervention also resulted in improved disclosure rates by caregivers to children about their HIV status, showing positive shifts in communication and trust.	Increased adherence, better retention in care, enhanced psychosocial well‐being, positive social support
2	Rout et al. (2019)	Observational, with a cross‐sectional and comparative approach	6 ART centers	Telemedicine initiative introduced in Maharashtra to improve treatment compliance for pediatric HIV‐infected patients. This initiative connects remote ART (antiretroviral therapy) centers to a Pediatric HIV Center of Excellence (PCoE) through video‐link for consultations, management of complex cases, counseling, and training of healthcare personnel.	The cost per‐visit in telemedicine‐linked centers was significantly lower (INR 1803) compared to nonlinked centers, telemedicine‐linked centers had a 5 percentage point improvement in lost‐to‐follow‐up cases, and the timeliness of patient visits improved, telemedicine contributed to greater efficiency in resource use, as the increased patient load reduced the average per‐visit cost. The telemedicine‐linked centers saw increased compliance with ART treatment protocols, showing better attendance and reduced visit intervals over time compared to nonlinked centers.	The telemedicine‐linked centers demonstrated cost savings and improved efficiency, with a decrease in per‐pediatric patient cost by INR 557 compared to nonlinked centers. Treatment compliance improved, as seen in the timeliness of patient visits and reduced follow‐up loss.
3	Manglani et al. (2022)	A qualitative research design	Caregivers 27 participants	Pediatric HIV telemedicine initiative. This video‐linked service connects pediatric HIV experts from the Pediatric Center of Excellence (PCoE) in Mumbai to remote ART centers.	High acceptance of telemedicine by children, caregivers, and healthcare providers; reduced travel burden and cost savings for families, as they no longer need to travel to distant referral centers for specialist consultations; healthcare providers benefitted from discussing complex cases with specialists and appreciated the inclusion of counselors and nutritionists during the TM sessions; group counseling sessions via telemedicine were found to be beneficial, where multiple families could engage in shared learning and peer support.	Telemedicine helped improve the geographical and financial access to pediatric HIV care, especially for patients from rural or remote areas; timely interventions through telemedicine improved clinical management, reducing lost follow‐up cases and potentially improving patient outcomes; caregivers felt that telemedicine reduced the economic and social strain on their families, as they could avoid the challenges of traveling to big cities like Mumbai.
4	Sánchez et al. (2021)	A randomized controlled trial	Adolescents, (13–18) 186 participants	Text message (SMS) reminder system	Willingness to receive SMS reminders: 81.1% of participants were willing to receive text message reminders; effectiveness of SMS reminders: The intervention group showed a 4% improvement in adherence over 6 months, while the control group showed no significant change (0.85%). Adolescents (13–18 years old) had the most significant improvement in adherence (5.5%)	SMS text message reminders were shown to significantly improve ART adherence in pediatric HIV‐infected patients, particularly adolescents. Participants in the intervention group had a 4‐fold higher chance of improving adherence compared to the control group.
5	Mtisi et al. (2024)	Retrospective observational cohort study	Adolescent and young adult (10–24), 107 participants	Pill counts, self‐reports, and DBS (dried blood spot) tenofovir concentrations.	Adherence levels varied across methods, with self‐reports showing the highest rates, while DBS measurements were more consistent	Participants who were adherent based on DBS concentrations were nearly six times more likely to achieve virological suppression
6	Swai et al. (2023)	A convergent parallel mixed‐methods design.	Children (0–14) 142 participants and adolescents (15–19) 142 participants	A 10‐item short adherence questionnaire to assess the level of ART adherence.	Most participants preferred receiving reminder SMS messages that were neutral and did not directly mention medication, to maintain confidentiality. Participants using the DAT found the adherence reports and reminders helpful, with 85% of survey participants expressing a desire to receive reminder SMS, and 80% preferring daily reminders	The intervention increased motivation to adhere to antiretroviral therapy, as the reminder system helped participants stay on track with their medication. Participants and their caregivers appreciated the feedback on medication adherence through graphs, which provided a clear understanding of their adherence behavior. The intervention demonstrated the feasibility of using digital tools to improve adherence in the target population

**Table 3:** Selected studies to discuss community‐based intervention, caregiver support program, and peer and community support.

No	Study	Design	Population	Intervention	Findings	Outcomes
1	Okonji et al. (2022)	A qualitative descriptive research design.	Adolescents (10–19), 173 participants	Psychosocial support (PSS) program delivered in public primary healthcare facilities.	The PSS program improved participants′ understanding of HIV and ART adherence through facilitated disclosure, health education, and peer support. It also enhanced their relationships with healthcare providers and provided emotional support. Adolescents reported better mental health, reduced feelings of isolation, and increased confidence in managing their HIV status	The program led to improved adherence to ART among participants, with many feeling more empowered and supported in their treatment journey. It also contributed to greater retention in care, even amid the challenges posed by the COVID‐19 pandemic
2	Griwmwood et al. (2012)	A multicenter cohort study	Children (< 16), 3563 participants	Community‐based adherence support provided by patient advocates (PAs) who deliver adherence and psychosocial support to children and caregivers	Retention: Children supported by PAs had significantly better retention rates after 3 years of ART (91.5%) compared to those without PA support (85.6%). Mortality: Corrected mortality after 3 years of ART was lower among PA‐supported children (3.7%) compared to nonsupported children (8.0%).	Retention in care: Community adherence support significantly improved retention in care, particularly for children under 2 years old. Reduced mortality: PA‐supported children had lower mortality rates, suggesting a protective effect of community‐based support.
3	Anígilájé et al. (2014)	Descriptive, longitudinal study with a qualitative focused group discussion.	Preschool children (0–5), preadolescent (6–8), adolescent (9–15), 33 participants	Kiddies′ Club (KC) providing psychosocial support, leisure activities, and education on adherence.	Adherence: 100% adherence to ART was observed at both 6 and 12 months.	Retention in care: The Kiddies’ Club (KC), which provided psychosocial support and leisure activities, effectively retained children in care. Clinical improvement: Significant improvements were seen in the children’s clinical, immunological, and virological outcomes. Community engagement: The KC encouraged caregivers to engage in cooking and discussing barriers to clinic attendance, such as financial constraints.
4	Nasuuna et al. (2019)	An exploratory qualitative study	Caregivers, 37 participants	Intensive adherence counseling (IAC) program	Challenges in attending and completing IAC sessions: Health system barriers: Long clinic queues, poor quality of counseling, unfavorable appointment times for schoolchildren, lack of appointment reminders, and early closure of counseling services. Challenges in supporting adherence to ART: Environmental factors: Caregivers working away from home, children’s school activities, and issues with drugs (bitter taste, frequent dosing). Financial factors: Lack of food and financial instability affected both adherence and overall care. Personal factors: Nondisclosure of HIV status to children, caregiver fatigue, stigma, and lack of family support. Psychological factors: Caregiver guilt (especially mothers who passed HIV to their children), anxiety about the child’s future, and abandonment by other family members. Child‐related factors: Treatment fatigue and peer influence negatively impacted adherence.	Psychosocial support: More frequent peer support groups and assistance with disclosing HIV status to children and family members. Economic empowerment: Vocational training, income‐generating activities, and support with school fees. Health system reforms: Improved counseling quality tailored to children, better appointment scheduling, and reduction of drug doses (e.g., long‐acting injections instead of daily pills).

5	Campbell et al. (2011)	A qualitative research approach		The role of community‐based interventions, improved health services (such as the provision of CD4 count machines), and NGO involvement in delivering educational programs, food aid, and social support to ensure ART adherence.	Community and social networks: The study highlights how a supportive community, which includes guardians, health workers, and NGOs, is crucial for encouraging adherence. Role of NGOs: NGOs provided essential resources such as education on HIV/AIDS, food aid, and counseling services, which further reduced stigma and supported adherence. Health services: The availability of ART and the improvement of health services, such as CD4 count machines and motivated healthcare workers, were critical factors in supporting adherence. These services improved trust in the health system and motivated both caregivers and nurses. Guardians’ role: Guardians played a key role as treatment partners, ensuring children adhered to medication schedules. Children’s participation: Older children, when aware of their condition, were active participants in their treatment, often reminding guardians about medication schedules.	Children’s participation: Older children, when aware of their condition, were active participants in their treatment, often reminding guardians about medication schedules.

6	Amzel et al. (2013)	A quantitative study		School‐based interventions to reduce HIV stigma and support YLH	Resilience and support, school choice, impact on ART adherence and mental health, HIV stigma drivers, and manifestations	HIV education targeting teachers, students, and the broader school community is seen as essential for stigma reduction. Educating students on the realities of HIV transmission and treatment outcomes can correct misconceptions and reduce discrimination. Structured medication policies and confidential HIV disclosure protocols in schools are proposed to ensure adherence and reduce stigma. In‐school psychosocial support, either through trusted adults or support groups, could help YLH manage their condition and maintain ART adherence while reducing the psychological burden of stigma.
7	Njuguna et al. (2023)	A descriptive phenomenological design	Caregivers, 24 participants	Psychosocial support intervention	Drivers and facilitators of HIV stigma in schools stem from misconceptions about HIV transmission, treatment, and long‐term health; stigma manifests through gossip, isolation, and loss of friendships, leading to poor adherence to antiretroviral therapy (ART) and mental health challenges; fear of stigma impacts YLH’s school choices, disclosure of their status, and their ability to seek necessary support.	The study indicates that fear of stigma leads to poor ART adherence, particularly among YLH in boarding schools, and contributes to poor mental health. Stigma also influences decisions such as whether to attend a day school or a boarding school, and it affects academic performance and social relationships. YLH resilience, HIV education, and school‐based psychosocial support were highlighted as key to improving outcomes.

### 2.2. Study Selection Process

The selection process followed narrative review principles, emphasizing breadth rather than a systematic approach. The initial search identified a broad range of studies, which were then screened by title and abstract for relevance to the topic of pediatric ART adherence. Full‐text articles were subsequently reviewed to assess their eligibility based on the inclusion criteria. Priority was given to studies exploring practical, real‐world interventions such as mobile health solutions, caregiver support programs, and community‐based strategies in LMICs.

**Figure 1:**
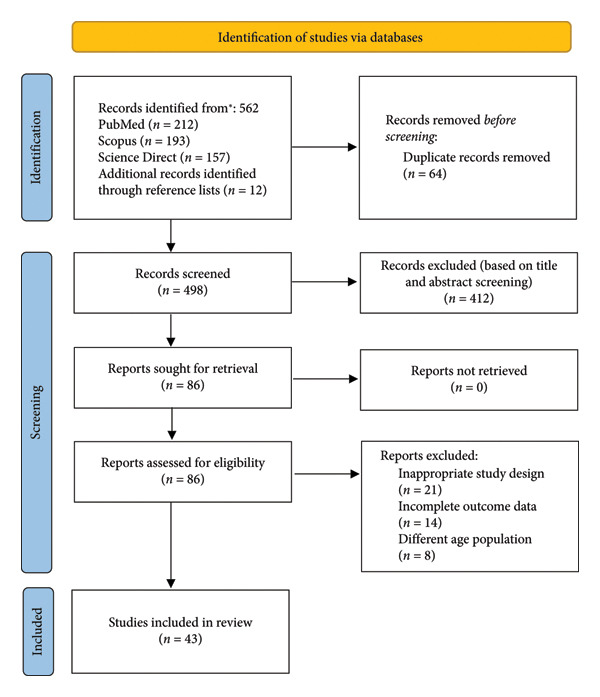
PRISMA flow diagram of study selection for this review.

Although no formal critical appraisal tools were applied, each included article was reviewed for methodological clarity and relevance to the review objectives. The authors assessed the studies based on the clarity of research aims, appropriateness of study design, adequacy of sample description, and relevance to pediatric ART adherence. Studies presenting clear methodology and contextually significant findings were prioritized for inclusion to ensure a balanced and representative synthesis of available evidence. The study selection process is summarized in Figure 1.

## 3. Results and Discussion

### 3.1. Factors Influencing ART Adherence

#### 3.1.1. Medication‐Related Factors—Contributing to Adherence

Medication‐related issues remain central to ART adherence in children, with formulation and regimen complexity being the most influential. The bitter taste of drugs such as zidovudine (AZT) and several protease inhibitors has been repeatedly cited as a major barrier, discouraging children from consistent use, while flavored or liquid formulations are better accepted and associated with higher adherence [17]. The physical characteristics of ART also play a role: Oversized tablets are difficult for children to swallow, often forcing caregivers to crush them or find alternative methods of administration, which complicates treatment and reduces adherence [17]. In contrast, smaller or liquid preparations facilitate easier administration and improve adherence [17].

Regimen complexity adds further challenges. Multipill schedules and regimens requiring frequent dosing increase caregiver burden and the likelihood of missed doses [17]. Efavirenz‐based regimens, in particular, have been linked to neuropsychiatric side effects that negatively affect adherence [17]. By comparison, FDCs simplify dosing, reduce pill burden, and consistently demonstrate higher adherence rates [18, 19].

Differences across specific drug regimens are also important. AZT‐based regimens are associated with more pronounced side effects, contributing to discontinuation and poorer adherence, whereas tenofovir (TDF)‐based regimens are generally better tolerated [8, 14]. Children on TDF not only achieve better viral suppression but also experience improved health outcomes, which reinforce adherence behaviors by providing psychological motivation for continued treatment [8, 14].

#### 3.1.2. Socioeconomic Factors and Family Dynamics

Socioeconomic conditions and family dynamics are critical determinants of ART adherence among children and adolescents. Children without stable parental figures or those who are orphans often face greater challenges in maintaining adherence, compounded by psychological stress and reduced confidence in treatment [17, 20]. Financial instability, limited access to healthcare, and transportation difficulties further hinder consistent treatment, especially in low‐income families [8, 14].

Family cohesion and communication strongly influence adherence outcomes. Adolescents who perceive their families as supportive and cohesive are more likely to report higher adherence, while poor communication and family conflict correlate with nonadherence [18, 19]. Open discussions about HIV and ART within the household foster responsibility and reduce stigma, whereas a lack of communication increases isolation and misunderstandings, leading to missed doses [17, 18].

Caregiver involvement remains central to pediatric adherence. Caregivers provide emotional, instrumental, informational, and appraisal support, ranging from daily reminders and medication management to accompanying children to clinic visits, all of which significantly improve adherence rates [7, 21]. Evidence from Uganda consistently shows that children supported by cohesive and engaged families are more likely to adhere to ART, underscoring the value of family‐based interventions [18–20].

#### 3.1.3. Caregiver‐Related Factors

Caregivers are central to pediatric ART adherence, as children are unable to manage treatment independently. Their knowledge and understanding of HIV and ART strongly influence outcomes: informed caregivers are more capable of supervising daily doses, recognizing side effects, and navigating healthcare systems [7, 8, 22]. Conversely, caregivers with limited knowledge are more likely to neglect medication schedules and follow‐up visits, leading to inconsistent adherence [22, 23].

The health and psychosocial status of caregivers also play an important role. Caregivers who are themselves unwell, depressed, or experiencing stigma‐related stress may struggle to provide consistent support [17, 24]. Stigma and fear of discrimination can further discourage them from attending clinics or disclosing the child’s HIV status, which undermines continuity of care [17].

Recent evidence from multicountry and regional studies further supports these findings. A 2025 analysis from the International Epidemiology Databases to Evaluate AIDS (IeDEA) network highlighted that adherence monitoring and support systems for children and adolescents vary widely across regions, with suboptimal viral suppression particularly among younger age groups. The study emphasized that programs incorporating structured caregiver engagement and digital adherence tracking showed the highest success rates in maintaining viral suppression among pediatric populations [25].

Similarly, Şensoy et al. (2025) identified that caregiver‐related stress, stigma, and health system barriers remain critical determinants of adherence among children living with HIV in Türkiye. Their qualitative findings reaffirm the global relevance of psychosocial and structural support, emphasizing that caregiver empowerment and consistent counseling play a pivotal role in sustaining long‐term ART adherence [26].

Education is a critical factor in caregiver capacity. Caregivers with formal education are better able to navigate health systems, understand the importance of treatment, and create a supportive environment that reduces stigma [18, 20, 23]. Evidence from Ethiopia shows that caregivers with education beyond the fifth grade are significantly more effective in ensuring consistent adherence among children [20].

Caregiver demographics also shape adherence. Maternal figures and female caregivers are generally more attentive to children’s ART routines, while younger or single caregivers often face greater stress and fewer resources, which negatively affect adherence [7, 8, 20].

Finally, the quality of the caregiver–child relationship and the level of family engagement amplify adherence outcomes. A positive, supportive relationship fosters trust and motivation in children, while conflict or neglect reduces adherence [18, 19]. Evidence from Uganda highlights that cohesive families and engaged caregivers through emotional, instrumental, informational, and appraisal support significantly improve adherence rates in children and adolescents [18, 19, 21, 22].

#### 3.1.4. Psychosocial and Adolescent‐Specific Factors

Psychosocial challenges are among the most persistent barriers to ART adherence in pediatric populations. Stigma remains a dominant issue: children and adolescents frequently experience discrimination from peers and community members, leading to secrecy around medication and reluctance to take ART in public [9, 10, 27]. Fear of disclosure not only undermines adherence but also contributes to psychological distress, including anxiety and depression [13, 14, 28].

Mental health is another critical factor. Depression, low self‐esteem, and poor coping skills are consistently linked to poor adherence [28–30]. Family conflict and lack of psychosocial support within households often compound these difficulties, creating an environment that fosters isolation and disengagement from care [14, 20, 27].

Adolescents face additional challenges as they assume greater responsibility for their treatment. Pill fatigue defined as the burden of taking medication daily over many years is commonly reported and contributes to intentional missed doses [10, 20, 31]. Peer pressure further exacerbates this issue, as adolescents may avoid taking medication at school or in front of friends out of fear of stigma, choosing social acceptance over consistent adherence [9, 10, 14]. The transition from pediatric to adult care represents another vulnerable period, as adolescents lose adolescent‐focused services and often face reduced family involvement, resulting in increased risk of treatment discontinuation [20, 31].

Despite these challenges, psychosocial interventions demonstrate measurable benefits. Counseling services, peer‐led programs, and community adolescent treatment supporters (CATS) have been shown to improve ART adherence, reduce stigma, and strengthen psychosocial well‐being among adolescents [30–32]. Continued provision of these interventions is critical to addressing the unique vulnerabilities of this age group.

#### 3.1.5. Healthcare‐Associated Barriers

Barriers within healthcare systems significantly affect pediatric ART adherence in LMICs. One of the most frequently reported challenges is drug stock‐outs, which disrupt continuity of treatment and force children and caregivers to skip doses or switch regimens, undermining viral suppression and adherence [13, 33, 34]. Inconsistent medication supply has been documented across multiple LMIC settings, highlighting the vulnerability of children to systemic resource shortages [33, 34].

Health service delivery limitations also reduce adherence. Long clinic wait times, understaffed facilities, and inadequate counseling services create barriers to consistent attendance [27, 33, 35]. Adolescents, in particular, often describe negative experiences with healthcare providers, including judgmental attitudes and lack of confidentiality, which deter regular visits and engagement with care [36, 37].

Accessibility challenges further compound these issues. Families in rural areas may struggle with long travel distances and high transportation costs, creating a major obstacle to routine clinic attendance [8, 22, 35]. These difficulties disproportionately affect poorer households and have been consistently linked to missed appointments and lower adherence levels [22, 35].

Healthcare infrastructure weaknesses also manifest in limited adolescent‐friendly services. Many systems lack programs tailored to the developmental needs of youth, and the absence of confidential, supportive environments discourages adolescents from seeking care consistently [20, 36, 37]. The transition from pediatric to adult services is especially problematic when adolescent‐specific support structures are absent, often leading to disengagement [20, 31].

Despite these challenges, evidence shows that health system interventions including community‐based delivery models, improved provider training, and integration of psychosocial support into clinic services can significantly enhance adherence outcomes [30, 32, 34]. Strengthening these structural supports is essential to sustaining adherence in pediatric populations, especially in resource‐limited contexts.

#### 3.1.6. Emerging Challenges

Beyond individual and health system factors, recent reductions in international donor funding for pediatric ART programs in LMICs, such as proposed > 6% cut in PEPFAR, the largest international donor program for HIV/AIDS, currently supports ART access for over 20 million people worldwide, including more than one million children, funding for 2025 have resulted in drug stock‐outs and treatment interruptions, undermining continuity of care [1, 38]. Modeling studies indicate that a permanent discontinuation of PEPFAR could lead to approximately 660.000 new HIV infections in children (2025–2029) and up to 300.000 additional child deaths [39].

### 3.2. Strategies to Improve Adherence on Pediatric HIV

#### 3.2.1. Policy Recommendations

Effective policy implementation plays a pivotal role in sustaining and improving ART adherence among children and adolescents. Several country‐level initiatives provide valuable models. In Ghana, the adoption of the Person‐Centered Care Assessment Tool (PCC‐AT) has enhanced the alignment of healthcare delivery with patients’ physical, psychological, and social needs, creating supportive environments that foster adherence and collaboration between patients and providers [36]. In Nigeria, the National HIV Strategy for Adolescents and Young People highlights the importance of integrating psychosocial, educational, and economic support into HIV programs to address stigma and discrimination, both of which are significant barriers to adherence [16].

Innovative health policies have also been developed in Tanzania, where telemedicine is promoted to expand ART access in rural settings. Telemedicine initiatives have reduced costs, improved continuity of care, and enhanced follow‐up engagement for pediatric HIV‐infected patients [40, 41]. These examples demonstrate that policy frameworks tailored to local contexts can effectively complement biomedical interventions.

At the same time, recent donor funding cuts underline the urgency of building resilient health systems. While international programs such as PEPFAR and the Global Fund remain essential, reliance on external donors poses risks to program stability. National governments must prioritize domestic resource mobilization, integrate pediatric HIV care into universal health coverage, and establish sustainable financing mechanisms to protect treatment continuity and adherence gains in LMICs [1, 36].

#### 3.2.2. Peer and Community Support

Peer and community‐based interventions provide measurable improvements in ART adherence among children and adolescents. In Zimbabwe, the *CATS* program, which engaged HIV‐positive peers as treatment supporters, increased viral suppression rates by 8%–13% and significantly improved adherence and psychosocial well‐being compared to standard care [32]. In South Africa, community adherence support improved long‐term program retention by approximately 12% at 24 months, reducing the risk of treatment interruptions in pediatric populations [34].

Peer‐led youth clubs and community psychosocial support groups also demonstrated strong impacts. In Uganda, adolescents participating in peer support programs achieved retention rates of 84% compared to 72% among nonparticipants [20]. Structured peer groups not only reduced stigma but also lowered rates of missed doses by 15%–20%, while improving clinic attendance by up to 10% [30, 42]. These findings highlight the central role of peer involvement and community‐based support structures in sustaining adherence and reducing psychosocial barriers in resource‐limited settings.

#### 3.2.3. Caregiver and Family Support

Caregivers and families are central to pediatric ART adherence because children depend on them for daily supervision and emotional support. Evidence from multiple LMICs highlights the strong influence of caregiver education, household stability, and family cohesion on treatment outcomes. In India, adherence rates reached 91.4% among children whose caregivers had completed at least primary education, compared to substantially lower rates among those with less educated caregivers [17]. Similarly, in Ethiopia, caregiver education and stable household support were associated with adherence levels of 83%–89%, underscoring the protective effect of socioeconomic stability [23].

In Uganda, family‐based interventions demonstrated substantial improvements. A structured family support program increased adolescent adherence from 66% to 83%, while cohesive family environments and open caregiver–adolescent communication were strong predictors of sustained adherence [18–20]. Maternal involvement and female caregivers in particular have been shown to foster higher adherence levels, whereas younger or single caregivers often face stress and resource limitations that hinder consistent treatment [7, 8, 20].

Beyond education and demographics, caregivers’ psychosocial health also matters. Caregivers experiencing stigma, depression, or poor health themselves often struggle to provide consistent supervision, contributing to higher rates of missed doses [22, 24]. Conversely, caregiver counseling and empowerment programs not only improved medication supervision but also reduced missed clinic visits and improved viral suppression in children [19, 20].

Collectively, these findings demonstrate that adherence is not solely an individual responsibility of the child but is deeply embedded within caregiver capacity and family dynamics. Interventions that strengthen caregiver knowledge, reduce their psychosocial burden, and provide structured family support are essential for sustaining ART adherence in pediatric populations.

#### 3.2.4. Community‐Based Health System Linkages

Strengthening health system linkages at the community level has proven effective in addressing structural barriers to ART adherence among children and adolescents. Community‐based delivery models, task‐shifting to community health workers, and decentralized ART services help reduce the burden of distance, cost, and clinic wait times, particularly for rural families.

In South Africa, community adherence support improved retention among pediatric cohorts by approximately 12% at 24 months compared to facility‐based care, demonstrating the value of shifting adherence support into communities [34]. Similarly, in Brazil, enhanced caregiver–clinic collaboration and community‐centered care models improved clinic attendance and adherence, with children in supportive care frameworks achieving higher viral suppression rates compared to standard care [33].

In Ethiopia, children attending hospitals with integrated adherence counseling and community outreach services demonstrated significantly better adherence compared to those receiving standard clinic visits alone [35]. Telemedicine initiatives further extend these benefits by reducing geographical barriers. In India, telemedicine models improved treatment compliance and reduced costs, enabling continued access for pediatric patients in resource‐limited settings [40]. In Tanzania, families reported that telemedicine platforms facilitated follow‐up care and improved continuity of treatment, especially in remote areas where transportation costs are prohibitive [41].

Adolescent‐friendly adaptations of community services are also critical. In Cambodia, adolescents receiving care in youth‐oriented ART clinics demonstrated improved engagement compared to standard adult‐oriented services [37]. These findings show that health system reforms are vital to improving both retention and adherence outcomes.

#### 3.2.5. Technological Innovations

Digital health innovations are increasingly recognized as effective tools to enhance ART adherence among children and adolescents in LMICs. Low‐cost, scalable technologies such as SMS reminders, mobile applications, and electronic adherence monitoring offer additional support for populations facing structural and psychosocial barriers.

SMS reminder systems have shown measurable though modest effects. A meta‐analysis of text messaging interventions found improvements in adherence of up to 5.5% among adolescents compared to standard care [12]. In Guatemala, a pediatric HIV clinic implementing text message reminders reported significant reductions in missed doses, with clinic adherence rates improving from 74% to 82% after 6 months of intervention.^43^

Customized adherence tools tailored to youth have demonstrated stronger results. In Tanzania, a digital adherence tool integrated into routine care improved both retention and adherence rates by approximately 10%–12%, confirming the value of personalized digital support.^11,44^ Similarly, electronic adherence monitoring combined with digital feedback loops has been associated with better viral suppression and higher adherence scores in pediatric populations.^44^

In Southeast Asia, where self‐reported adherence rates among adolescents were often below 80%, the introduction of Audio Computer‐Assisted Self‐Interview (ACASI) systems helped identify high‐risk patients and facilitated targeted counseling, contributing to improved treatment monitoring and adherence support [10]. Collectively, these findings highlight that digital interventions, while not a panacea, provide consistent incremental improvements in adherence. When embedded within community and family support systems, technological solutions amplify treatment outcomes and reduce the risk of disengagement among children and adolescents living with HIV.

## 4. Conclusions

Adherence to ART among children and adolescents in LMICs remains suboptimal, yet consistent improvements are observed through family‐centered interventions, caregiver support, community engagement, and digital health tools. However, these gains are fragile, as funding reductions, transition challenges from pediatric to adult care, and adherence fatigue continue to threaten long‐term outcomes. Sustaining progress will require integrated approaches that combine simplified regimens, psychosocial and community‐based support, and resilient financing policies to ensure continuity of care and improved health for pediatric and adolescent populations.

## Conflicts of Interest

The authors declare no conflicts of interest.

## Funding

This research received no external funding.

## Data Availability

No new data were created or analyzed in this study. Data sharing is not applicable to this article.

## References

[1] UNAIDS , Global AIDS Report—the Urgency of Now: AIDS at a Crossroads; 2024, 2024, http://www.wipo.int/.

[2] Mar’ah S. S. , Ichsan B. , and Cholisoh Z. , Demographic, Clinical, and Antiretroviral Regiment Treatment Characteristics of HIV/AIDS Patients at the Regional General Hospital of Central Papua, Pharma: Jurnal Farmasi Indonesia. (2023) 20, no. 2, 178–185.

[3] UNICEF , Global and Regional Trends: HIV/AIDS Data, 2025, UNICEF Data, https://data.unicef.org/topic/hivaids/global-regional-trends/.

[4] Brittain K. , Asafu-Agyei N. A. , Hoare J. et al., Association of Adolescent- and Caregiver-Reported Antiretroviral Therapy Adherence with HIV Viral Load Among Perinatally-Infected South African Adolescents, AIDS and Behavior. (2018) 22, no. 3, 909–917, 10.1007/s10461-017-2004-2, 2-s2.0-85037613820. 29224045 PMC6620475

[5] Gemechu G. B. , Hebo H. , and Kura Z. , Children’s Adherence to Antiretroviral Therapy and Associated Factors: Multicenter Cross-Sectional Study, HIV. (2023) 15, 423–434, 10.2147/HIV.S407105. PMC10368110 37497118

[6] Kurniawati V. V. , Harioputro D. R. , and Susanto A. J. , Evaluation of CD4 Cell Count, Viral Load, and Neutrophil-Lymphocyte Ratio (NLR) on Opportunistic Infections in HIV/AIDS Patients, Biomedika. (2022) 14, no. 2, 99–107, 10.23917/biomedika.v14i2.17299.

[7] Lahai M. , James P. B. , Wannang N. N. M. et al., A Cross-Sectional Study on Caregivers’ Perspective of the Quality of Life and Adherence of Paediatric HIV Patients to Highly Active Antiretroviral Therapy, BMC Pediatrics. (2020) 20, no. 1, 10.1186/s12887-020-02194-7. PMC7282047 32517722

[8] Mengesha M. M. , Embibel M. , Gobena T. , Tunje A. , Jerene D. , and Hallström I. K. , Antiretroviral Therapy Non-adherence Among Children Living with HIV in Dire Dawa, Eastern Ethiopia: a case-control Study, BMC Pediatrics. (2022) 22, no. 1, 10.1186/s12887-022-03697-1. PMC9647744 36357856

[9] Utami I. T. , Prakoeswa F. R. S. , Lestari N. , and Ichsan B. , Relationship Between Knowledge Level and Community Stigma Toward HIV/AIDS Infection in Indonesia: a Literature Review, Jurnal Kedokteran Syiah Kuala. (2023) 23, no. 1, 99–107, 10.24815/jks.v23i1.24678.

[10] Prasitsuebsai W. , Sethaputra C. , Lumbiganon P. et al., Adherence to Antiretroviral Therapy, Stigma and Behavioral Risk Factors in HIV-Infected Adolescents in Asia, AIDS Care. (2018) 30, no. 6, 727–733, 10.1080/09540121.2018.1425363, 2-s2.0-85043394130. 29336591 PMC5912939

[11] Sumari-de Boer I. M. , Ngowi K. M. , Swai I. U. et al., Effect of a Customized Digital Adherence Tool on Retention in Care and ART Adherence in Breastfeeding Women, Children and Adolescents in Tanzania: a Mixed-Methods Study Followed by Clinical Trials, Trials. (2023) 24. 10.1186/s13063-023-07293-1 PMC10120095 37085913

[12] Aristya P. F. , Prasetya H. , and Ichsan B. , The Effectiveness of Mobile Phone Text Messages on ART Adherence in PLHIV: a Meta-Analysis, J Health Promotion and Behavior. (2023) 8, no. 3, 195–205.

[13] Martelli G. , Antonucci R. , Mukurasi A. , Zepherine H. , and Nöstlinger C. , Adherence to Antiretroviral Treatment Among Children and Adolescents in Tanzania: Comparison Between Pill Count and Viral Load Outcomes in a Rural Context of Mwanza Region, PLoS One. (2019) 14, no. 3, 10.1371/journal.pone.0214014, 2-s2.0-85063336942. PMC6428300 30897131

[14] Marta Castro M. D. M. S. , Ida González M. D. PhD. , and Jorge Pérez M. D. M. S. , Factors Related to Antiretroviral Therapy Adherence in Children and Adolescent with HIVAIDS in Cuba, MEDICC Rev. (2015) 17, no. 1, 35–40. 25725767 10.37757/MR2015.V17.N1.8

[15] Nichols J. S. , Kyriakides T. C. , Antwi S. et al., High Prevalence of Non-adherence to ART Among Undisclosed HIV-Infected Children in Ghana, AIDS Care. (2019) 31, no. 1, 25–34, 10.1080/09540121.2018.1524113, 2-s2.0-85053662469. 30235940 PMC6288009

[16] National Agency for The Control of AIDS , National HIV Strategy for Adolescents and Young People 2016-2022, 2016.

[17] Bhattacharya M. and Dubey A. P. , Adherence to Antiretroviral Therapy and its Correlates Among HIV-infected Children at an HIV Clinic in New Delhi, Annals of Tropical Paediatrics. (2011) 31, no. 4, 331–337, 10.1179/1465328111Y.0000000031, 2-s2.0-80055030629. 22041467

[18] Nabunya P. , Samuel K. , and Ssewamala F. M. , The Effect of Family Support on self-reported Adherence to ART Among Adolescents Perinatally Infected with HIV in Uganda: a Mediation Analysis, Journal of Adolescence. (2023) 95, no. 4, 834–843, 10.1002/jad.12157. 36810778 PMC10257769

[19] Nabunya P. , Bahar O. S. , Chen B. , Dvalishvili D. , Damulira C. , and Ssewamala F. M. , The Role of Family Factors in Antiretroviral Therapy (ART) Adherence self-efficacy Among HIV-infected Adolescents in Southern Uganda, BMC Public Health. (2020) 20, no. 1, 10.1186/s12889-020-8361-1. PMC7077174 32183762

[20] Nabukeera-Barungi N. , Elyanu P. , Asire B. et al., Adherence to Antiretroviral Therapy and Retention in Care for Adolescents Living with HIV from 10 Districts in Uganda, BMC Infectious Diseases. (2015) 15, no. 1, 10.1186/s12879-015-1265-5, 2-s2.0-84946909857. PMC4647509 26573923

[21] Bermudez L. G. , Jennings L. , Ssewamala F. M. , Nabunya P. , Mellins C. , and McKay M. , Equity in Adherence to Antiretroviral Therapy Among Economically Vulnerable Adolescents Living with HIV in Uganda, AIDS Care-Psychological and Socio-Medical Aspects of AIDS/HIV. (2016) 28, no. sup2, 83–91, 10.1080/09540121.2016.1176681, 2-s2.0-84978134372. PMC4940111 27392003

[22] Vreeman R. C. , Scanlon M. L. , Tu W. , Slaven J. , Ayaya S. , and Nyandiko W. , Validation of a Short Adherence Questionnaire for Children Living with HIV on Antiretroviral Therapy in Kenya, Journal of the International Association of Physicians in AIDS Care. (2018) 17, 10.1177/2325958218820329, 2-s2.0-85059157434.

[23] Eticha T. and Berhane L. , Caregiver-Reported Adherence to Antiretroviral Therapy Among HIV Infected Children in Mekelle, Ethiopia, BMC Pediatrics. (2014) 14, no. 1, 10.1186/1471-2431-14-114, 2-s2.0-84900034949. PMC4018187 24766911

[24] Yiryuo L. , Kpekura S. , Osman W. et al., Challenges and Support Experienced by Family Caregivers Seeking Antiretroviral Therapy Services for Children Living with HIV/AIDS: a Phenomenological Study in Ghana, BMJ Open. (2024) 14, no. 5, 10.1136/bmjopen-2023-081036. PMC11103234 38760044

[25] Nasuuna E. , Kigozi J. , Muwanguzi P. A. et al., Challenges Faced by Caregivers of Virally Non-suppressed Children on the Intensive Adherence Counselling Program in Uganda: a Qualitative Study, BMC Health Services Research. (2019) 19, no. 1, 10.1186/s12913-019-3963-y, 2-s2.0-85062622880. PMC6407183 30845951

[26] Anígilájé E. A. , Dabit O. J. , Tyovenda R. K. et al., Effects of Leisure Activities and Psychosocial Support on Medication Adherence and Clinic Attendance Among Children on Antiretroviral Therapy, HIV. (2014) 6, 127–137, 10.2147/HIV.S64964, 2-s2.0-84907909782. PMC4155831 25210476

[27] Smith F. M. C. , Eustache E. , Oswald C. et al., Psychosocial Support Intervention for HIV-Affected Families in Haiti: Implications for Programs and Policies for Orphans and Vulnerable Children, Social Science & Medicine. (2012) 74, no. 10, 1494–1503, 10.1016/j.socscimed.2012.01.022, 2-s2.0-84859759656. 22444459

[28] Okonji E. F. , Wyk B. v. , Hughes G. D. , and Mukumbang F. C. , Psychosocial Support Programme Improves Adherence and Health Systems Experiences for Adolescents on Antiretroviral Therapy in Mpumalanga Province, South Africa, International Journal of Environmental Research and Public Health. (2022) 19, no. 23, 10.3390/ijerph192315468. PMC9739873 36497544

[29] Mtisi T. J. , Kouamou V. , Morse G. D. , Dzinamarira T. , and Ndhlovu C. E. , Comparing Pill Counts and Patient self-reports Versus DBS Tenofovir Concentrations as ART Adherence Measurements with Virologic Outcomes and HIV Drug Resistance in a Cohort of Adolescents and Young Adults Failing ART in Harare, Zimbabwe, Journal of Infection and Public Health. (2024) 17, no. 9, 10.1016/j.jiph.2024.102500. PMC11393767 39173560

[30] Willis N. , Milanzi A. , Mawodzeke M. et al., Effectiveness of Community Adolescent Treatment Supporters (CATS) Interventions in Improving Linkage and Retention in Care, Adherence to ART and Psychosocial well-being: a Randomised Trial Among Adolescents Living with HIV in Rural Zimbabwe, BMC Public Health. (2019) 19, no. 1, 10.1186/s12889-019-6447-4, 2-s2.0-85060625264. PMC6348677 30691425

[31] Ricci G. , Netto E. M. , Luz E. , Rodamilans C. , and Brites C. , Adherence to Antiretroviral Therapy of Brazilian HIV-Infected Children and Their Caregivers, Brazilian Journal of Infectious Diseases. (2016) 20, no. 5, 429–436, 10.1016/j.bjid.2016.05.009, 2-s2.0-84991199477. PMC9425490 27471126

[32] Grimwood A. , Fatti G. , Mothibi E. , Malahlela M. , Shea J. , and Eley B. , Community Adherence Support Improves Programme Retention in Children on Antiretroviral Treatment: a Multicentre Cohort Study in South Africa, Journal of the International AIDS Society. (2012) 15, no. 2, 10.7448/IAS.15.2.17381, 2-s2.0-84867471904. PMC3499784 22713255

[33] Arage G. , Tessema G. A. , and Kassa H. , Adherence to Antiretroviral Therapy and Its Associated Factors Among Children at South Wollo Zone Hospitals, Northeast Ethiopia: a cross-sectional Study, BMC Public Health. (2014) 14, no. 1, 10.1186/1471-2458-14-365, 2-s2.0-84899942091. PMC4002537 24735561

[34] Posner J. E. , Duffy M. , Madevu-Matson C. et al., Do HIV Provider and Client Perspectives Align on person-centered Care? Lessons Learned from Implementation of the Person-Centered Care Assessment Tool (PCC-AT) in HIV Treatment Settings in Ghana, PLOS Global Public Health. (2024) 4, no. 9, 10.1371/journal.pgph.0003457. PMC11379259 39240928

[35] Yi S. , Tuot S. , Pal K. et al., Characteristics of Adolescents Living with HIV Receiving Care and Treatment Services in Antiretroviral Therapy Clinics in Cambodia: Descriptive Findings from a cross-sectional Study, BMC Health Services Research. (2018) 18, no. 1, 10.1186/s12913-018-3580-1, 2-s2.0-85054995116. PMC6192163 30326882

[36] Biden Administration Plans Major Cuts to AIDS Relief Programs in Africa-POLITICO, https://www.politico.com/news/2024/07/02/biden-administration-cut-aids-relief-africa-00166298?utm_source=chatgpt.com.

[37] Brink D. T. , Martin-Hughes R. , Bowring A. L. et al., Impact of an International HIV Funding Crisis on HIV Infections and Mortality in low-income and middle-income Countries: a Modelling Study, Lancet HIV. (2025) 12, no. 5, e346–e354, 10.1016/S2352-3018(25)00074-8. 40157378

[38] Rout S. K. , Gabhale Y. R. , Dutta A. et al., Can Telemedicine Initiative Be an Effective Intervention Strategy for Improving Treatment Compliance for Pediatric HIV Patients: Evidences on Costs and Improvement in Treatment Compliance from Maharashtra, India, PLoS One. (2019) 14, no. 10, 10.1371/journal.pone.0223303, 2-s2.0-85073052488. PMC6782091 31593580

[39] Manglani M. , Lala M. M. , Gabhale Y. et al., Attitudes and Acceptability of Children, Caregivers, and Healthcare Providers About Using Telemedicine for Pediatric HIV Care in a resource-limited Setting, PLoS One. (2022) 17, no. 5 May, 10.1371/journal.pone.0268740. PMC9132320 35613106

[40] Amzel A. , Toska E. , Lovich R. et al., Promoting a Combination Approach to Paediatric HIV Psychosocial Support, AIDS. (2013) 27, no. Suppl 2, S147–S157, 10.1097/qad.0000000000000098. 24361624 PMC4672375

[41] Sánchez S. A. , Ramay B. M. , Zook J. et al., Toward Improved Adherence: a Text Message Intervention in an Human Immunodeficiency Virus Pediatric Clinic in Guatemala City, Medicine (Baltimore). (2021) 100, no. 10, 10.1097/MD.0000000000024867. PMC7969223 33725842

[42] Swai I. U. , Ten Bergen L. L. , Mtenga A. et al., Developing Contents for a Digital Adherence Tool: a Formative mixed-methods Study Among Children and Adolescents Living with HIV in Tanzania, PLOS Digital Health. (2023) 2, no. 10, 10.1371/journal.pdig.0000232. PMC10584100 37851616

